# 3-Amino-5-methyl-5-(4-pyrid­yl)hydantoin

**DOI:** 10.1107/S1600536809011404

**Published:** 2009-04-02

**Authors:** Hristo Varbanov, Rossen Buyukliev, Adriana Bakalova, Alexander Roller

**Affiliations:** a Institute of Inorganic Chemistry, University of Vienna, Waehringerstrasse 42, A-1090 Vienna, Austria; b Department of Organic Chemistry, Faculty of Pharmacy, Medical University – Sofia, 2 Dunav Str., 1000 Sofia, Bulgaria; c Department of Chemistry, Faculty of Pharmacy, Medical University – Sofia, 2 Dunav Str., 1000 Sofia, Bulgaria

## Abstract

The title compound, 3-amino-5-methyl-5-(4-pyrid­yl)imid­azol­idine-2,4-dione, C_9_H_10_N_4_O_2_, was obtained by reaction of 5-methyl-5-(4-pyrid­yl)hydantoin with hydrazine. It crystallizes as a racemate in the tetra­gonal space group *I*4_1_/*a* with one mol­ecule in the asymmetric unit. The dihedral angle between the pyridine ring and the five-membered hydantoin ring is 47.99 (3)° In the crystal structure, mol­ecules are joined in a three-dimensional hydrogen-bonded network by N—H⋯N and N—H⋯O links.

## Related literature

For the biological activity of hydantoin derivatives and their metal complexes, see: Rajic *et al.* (2006 ▶); Bazil *et al.* (1998 ▶); Bakalova *et al.* (2005 ▶, 2008 ▶, 2009 ▶). For crystal structures of other 3-amino substituted hydantoins and their metal complexes, see: Shivachev *et al.* (2005 ▶); Bakalova *et al.* (2007 ▶). For the synthesis of 5-methyl-5-(4-pyridyl)-hydantoin, see: Chu & Teague (1958 ▶). For the preparation of 3-amino­hydantoins, see: Davidson (1964 ▶).
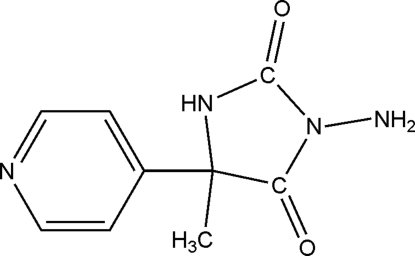


## Experimental

### 

#### Crystal data

C_9_H_10_N_4_O_2_
*M* *_r_* = 206.21
Tetragonal, 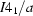

*a* = 12.8282 (5) Å
*c* = 22.9016 (17) Å
*V* = 3768.8 (3) Å^3^
*Z* = 16
Mo *K*α radiation
μ = 0.11 mm^−1^
*T* = 100 K
0.50 × 0.50 × 0.50 mm

#### Data collection

Bruker X8 APEXII CCD diffractometer
Absorption correction: multi-scan (*SADABS*; Bruker, 2005 ▶) *T*_min_ = 0.948, *T*_max_ = 0.948
51879 measured reflections
2765 independent reflections
2179 reflections with *I* > 2σ(*I*)
*R*_int_ = 0.076

#### Refinement

*R*[*F* ^2^ > 2σ(*F* ^2^)] = 0.046
*wR*(*F* ^2^) = 0.127
*S* = 1.03
2765 reflections
144 parameters
H atoms treated by a mixture of independent and constrained refinement
Δρ_max_ = 0.56 e Å^−3^
Δρ_min_ = −0.44 e Å^−3^

### 

Data collection: *APEX2* (Bruker, 2005 ▶); cell refinement: *SAINT* (Bruker, 2005 ▶); data reduction: *SAINT*; program(s) used to solve structure: *SHELXS97* (Sheldrick, 2008 ▶); program(s) used to refine structure: *SHELXL97* (Sheldrick, 2008 ▶); molecular graphics: *SHELXTL* (Sheldrick, 2008 ▶); software used to prepare material for publication: *SHELXTL*.

## Supplementary Material

Crystal structure: contains datablocks I, global. DOI: 10.1107/S1600536809011404/at2752sup1.cif

Structure factors: contains datablocks I. DOI: 10.1107/S1600536809011404/at2752Isup2.hkl

Additional supplementary materials:  crystallographic information; 3D view; checkCIF report

## Figures and Tables

**Table 1:** Hydrogen-bond geometry (Å, °)

*D*—H⋯*A*	*D*—H	H⋯*A*	*D*⋯*A*	*D*—H⋯*A*
N2—H2⋯N4^i^	0.88	2.69	3.526	159
N2—H2⋯O2^i^	0.88	2.26	2.9184 (15)	131
N4—H4*A*⋯O1^ii^	0.977 (19)	2.139 (19)	3.0676 (16)	158.07
N4—H4*B*⋯O1^iii^	0.96 (2)	2.17 (2)	3.1003 (16)	162.23

Symmetry codes: (i) 
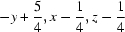
; (ii) 
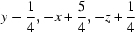
; (iii) 
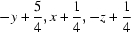
.

**Fig. 1.**
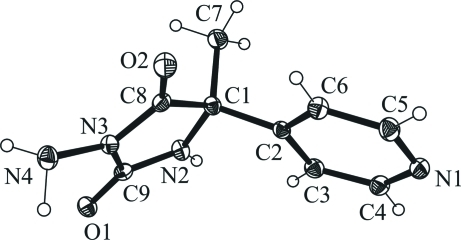
View of the molecule of AMPH with atom labeling scheme; the thermal ellipsoids are drawn at 50% probability level.

**Fig. 2.**
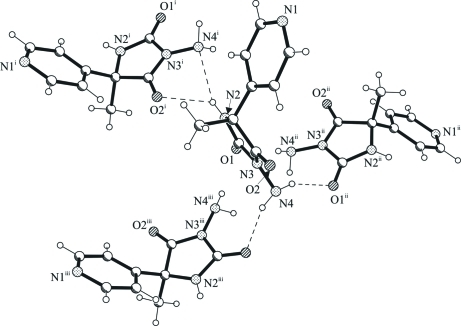
Fragment of the crystal structure of AMPH showing the intermolecular hydrogen bonding interactions. [Symmetry codes: (i) –y +1.25, x – 0.25, z – 0.25; (ii) y – 0.25, –x + 1.25, –z + 0.25; (iii) –y +1.25, x + 0.25, –z + 0.25].

## supplementary crystallographic information

### Comment

Some hydantoin derivatives are biologically active molecules with anticonvulsive, antiarythmic, antimicrobial, antiviral or cytostatic activitiy (Rajic *et al.*, 2006; Bazil *et al.*, 1998). 5-Methyl-5-(4-pyridyl)hydantoin was synthesized by Chu (Chu *et al.* 1958). This compound was used as starting material for preparation of new 3-amino-5-methyl-5-(4-pyridyl)hydantoin (AMPH). These hydantoin derivatives were utilized as carrier ligands for synthesis of new platinum and palladium complexes with potential cytotoxic activity. (Bakalova *et al.*, 2008, 2009). In the recent work we synthesized AMPH (I) by the method of Davidson (Davidson, 1964) with some modifications. The new compound was characterized by elemental analysis, IR, ^1^H and ^13^C NMR spectroscopy and molar conductivity. Suitable crystals of AMPH for X-ray diffraction analysis have been isolated and its structure was determined. The result of X-ray diffraction study of 3-amino-5-methyl-5-(4-pyridyl)hydantoin is shown in Fig. 1. The racemic compound crystallizes in the tetragonal space group *I*4_1_/*a* with one molecule in the asymmetric unit. The presence of the *sp*^3^-hybridized chiral carbon atom C1 is responsible for the dihedral angle between the pyridine ring and five-membered C_3_N_2_ ring of *ca* 48°. The sum of the angles around N4 is clearly smaller than 360° (327.0°), indicating its trigonal-pyramidal configuration. The lone-pair region at N4 is directed towards adjacent atom O1. The secondary amine nitrogen N2 acts as a proton donor in an intermolecular bifurcated hydrogen bonding interactions with the nitrogen atom N4 and oxygen atom O2 of the neighbouring molecule of AMPH (Fig. 2) The hydrazinic atom N4 is involved in two intermolecular H bonds with the atoms O1^ii^ and O1^iii^ of the two different neighbouring molecules (Table 1).

### Experimental

3-Amino-5-methyl-5-(4-pyridyl)hydantoin was synthesized by dissolving 5-methyl-5(4-pyridyl)hydantoin (1.91 g, 10 mmol) in 98% N_2_H_4_.H_2_O (5 *cm*^3^) and refluxing the solution for 2 h. The reaction mixture was cooled to room temperature and water (15 *cm*^3^) was added. The solution was placed in refrigerator for 24 h. The white product was filtered off, recrystallized from ethanol and dried at 373 K for 5 h. The purity was checked by TLC. The substance is soluble in DMSO and weakly soluble in water and ethanol. Yield: 1.26 g, 61%, m.p. = 523.2–524.7 K. Crystals, suitable for X-ray data collection were grown by slow evaporation from ethanol solution at 277 K.  Analysis calculated for C_9_H_10_N_4_O_2_: C 52.42, H 4.89, N 27.17%. Found: C 52.23, H 4.46, N 26.83%. λ_M_ = 0.979 S.*cm*^2^.mol^-1^; IR(pellets KBr)/cm^-1^: 3314.0, 3276.0, 1766.9, 1713.0, 1597.2 and 1411.1. ^1^H NMR (250 MHz; DMSO-d^6^): 8.95 (1*H*, s, N(1)—H), 8.59 (2*H*, d, J=7 Hz, H-2, H-6), 7.50 (2*H*, d, J=7 Hz, H-3, H-5), 4.80 (2*H*, s, NH2), 1.66 (3*H*, s, CH_3_). ^13^C NMR (62.5 MHz; DMSO-d^6^): 172.7 (C=O-4`), 155.5 (C=O-2`), 150.0 (C-2,*C*-6), 148.4 (C-4), 120.6 (C-3, C-5), 60.8 (C-5`), 24.7 (CH_3_).

### Refinement

H atoms were placed at calculated positions [N—H = 0.88 Å, C—H = 0.95 and 0.98 Å] and refined as riding atoms in the subsequent least squares model refinements, except two hydrogen atoms at N4 which were localized from difference map. The isotropic thermal parameters of hydrogen atoms in the positions of which were calculated were estimated to be 1.2 or 1.5 times the values of the equivalent isotropic thermal parameters of the atoms to which H atoms were bonded.

### Crystal data

**Table:**

C_9_H_10_N_4_O_2_	*D*_x_ = 1.454 Mg m^−^^3^
*M**_r_* = 206.21	Mo *K*α radiation, λ = 0.71073 Å
Tetragonal, *I*4_1_/*a*	Cell parameters from 847 reflections
Hall symbol: -I 4ad	θ = 2.9–29.5°
*a* = 12.8282 (5) Å	µ = 0.11 mm^−^^1^
*c* = 22.9016 (17) Å	*T* = 100 K
*V* = 3768.8 (3) Å^3^	Block, colourless
*Z* = 16	0.50 × 0.50 × 0.50 mm
*F*(000) = 1728	

### Data collection

**Table:**

Bruker X8 APEXII CCD diffractometer	2765 independent reflections
Radiation source: fine-focus sealed tube	2179 reflections with *I* > 2σ(*I*)
graphite	*R*_int_ = 0.076
ω scans	θ_max_ = 30.1°, θ_min_ = 2.9°
Absorption correction: multi-scan (*SADABS*; Bruker, 2005)	*h* = −18→18
*T*_min_ = 0.948, *T*_max_ = 0.948	*k* = −18→18
51879 measured reflections	*l* = −32→32

### Refinement

**Table:**

Refinement on *F*^2^	Primary atom site location: structure-invariant direct methods
Least-squares matrix: full	Secondary atom site location: difference Fourier map
*R*[*F*^2^ > 2σ(*F*^2^)] = 0.046	Hydrogen site location: inferred from neighbouring sites
*wR*(*F*^2^) = 0.127	H atoms treated by a mixture of independent and constrained refinement
*S* = 1.03	*w* = 1/[σ^2^(*F*_o_^2^) + (0.0631*P*)^2^ + 3.9566*P*] where *P* = (*F*_o_^2^ + 2*F*_c_^2^)/3
2765 reflections	(Δ/σ)_max_ < 0.001
144 parameters	Δρ_max_ = 0.56 e Å^−^^3^
0 restraints	Δρ_min_ = −0.44 e Å^−^^3^

### Special details

**Table:**

Geometry. All e.s.d.'s (except the e.s.d. in the dihedral angle between two l.s. planes) are estimated using the full covariance matrix. The cell e.s.d.'s are taken into account individually in the estimation of e.s.d.'s in distances, angles and torsion angles; correlations between e.s.d.'s in cell parameters are only used when they are defined by crystal symmetry. An approximate (isotropic) treatment of cell e.s.d.'s is used for estimating e.s.d.'s involving l.s. planes.
Refinement. Refinement of *F*^2^ against ALL reflections. The weighted *R*-factor *wR* and goodness of fit *S* are based on *F*^2^, conventional *R*-factors *R* are based on *F*, with *F* set to zero for negative *F*^2^. The threshold expression of *F*^2^ > σ(*F*^2^) is used only for calculating *R*-factors(gt) *etc*. and is not relevant to the choice of reflections for refinement. *R*-factors based on *F*^2^ are statistically about twice as large as those based on *F*, and *R*- factors based on ALL data will be even larger.

### Fractional atomic coordinates and isotropic or equivalent isotropic displacement parameters (Å^2^)

**Table:**

	*x*	*y*	*z*	*U*_iso_*/*U*_eq_	
O1	0.59104 (8)	0.59567 (7)	0.06315 (4)	0.0171 (2)	
O2	0.68145 (8)	0.42948 (8)	0.23252 (4)	0.0203 (2)	
N1	0.58287 (9)	0.06249 (9)	0.13055 (5)	0.0176 (2)	
N2	0.67304 (9)	0.43740 (9)	0.07949 (5)	0.0150 (2)	
H2	0.6820	0.4172	0.0431	0.018*	
N3	0.62479 (8)	0.53105 (8)	0.15592 (5)	0.0128 (2)	
N4	0.57993 (10)	0.61113 (9)	0.18907 (5)	0.0181 (2)	
H4A	0.5062 (15)	0.6163 (15)	0.1787 (8)	0.027 (5)*	
H4B	0.6100 (16)	0.6781 (17)	0.1806 (9)	0.036 (5)*	
C1	0.70691 (10)	0.37686 (10)	0.12997 (5)	0.0136 (3)	
C2	0.65758 (10)	0.26869 (10)	0.13127 (5)	0.0131 (2)	
C3	0.63039 (11)	0.21921 (10)	0.07925 (6)	0.0165 (3)	
H3	0.6365	0.2547	0.0430	0.020*	
C4	0.59419 (11)	0.11717 (11)	0.08121 (6)	0.0178 (3)	
H4	0.5764	0.0843	0.0454	0.021*	
C5	0.60863 (11)	0.11133 (11)	0.18034 (6)	0.0179 (3)	
H5	0.6008	0.0742	0.2160	0.021*	
C6	0.64606 (11)	0.21278 (10)	0.18292 (6)	0.0165 (3)	
H6	0.6635	0.2435	0.2194	0.020*	
C7	0.82617 (11)	0.36654 (11)	0.13212 (7)	0.0201 (3)	
H7A	0.8578	0.4360	0.1312	0.030*	
H7B	0.8502	0.3262	0.0984	0.030*	
H7C	0.8466	0.3308	0.1682	0.030*	
C8	0.67014 (10)	0.44623 (10)	0.18059 (6)	0.0139 (3)	
C9	0.62667 (10)	0.52740 (10)	0.09462 (5)	0.0132 (3)	

### Atomic displacement parameters (Å^2^)

**Table:**

	*U*^11^	*U*^22^	*U*^33^	*U*^12^	*U*^13^	*U*^23^
O1	0.0197 (5)	0.0162 (5)	0.0154 (5)	0.0016 (4)	−0.0004 (3)	0.0034 (3)
O2	0.0299 (6)	0.0178 (5)	0.0131 (5)	0.0007 (4)	−0.0040 (4)	0.0002 (4)
N1	0.0152 (5)	0.0153 (5)	0.0223 (6)	−0.0003 (4)	0.0004 (4)	0.0004 (4)
N2	0.0195 (5)	0.0145 (5)	0.0109 (5)	0.0020 (4)	0.0019 (4)	0.0013 (4)
N3	0.0136 (5)	0.0132 (5)	0.0117 (5)	0.0002 (4)	0.0011 (4)	−0.0005 (4)
N4	0.0194 (6)	0.0169 (6)	0.0180 (6)	0.0016 (4)	0.0023 (4)	−0.0030 (4)
C1	0.0153 (6)	0.0130 (6)	0.0126 (6)	0.0006 (4)	0.0000 (4)	0.0010 (4)
C2	0.0114 (5)	0.0127 (6)	0.0151 (6)	0.0014 (4)	0.0006 (4)	0.0005 (4)
C3	0.0177 (6)	0.0172 (6)	0.0146 (6)	0.0010 (5)	−0.0012 (5)	0.0008 (5)
C4	0.0178 (6)	0.0172 (6)	0.0183 (6)	0.0000 (5)	−0.0019 (5)	−0.0027 (5)
C5	0.0193 (6)	0.0162 (6)	0.0182 (6)	−0.0008 (5)	0.0013 (5)	0.0031 (5)
C6	0.0189 (6)	0.0160 (6)	0.0145 (6)	−0.0008 (5)	0.0005 (5)	0.0006 (5)
C7	0.0140 (6)	0.0175 (6)	0.0286 (7)	−0.0002 (5)	0.0006 (5)	0.0027 (5)
C8	0.0140 (6)	0.0134 (6)	0.0143 (6)	−0.0025 (4)	−0.0013 (4)	−0.0001 (4)
C9	0.0120 (6)	0.0151 (6)	0.0126 (6)	−0.0019 (5)	0.0011 (4)	0.0004 (4)

### Geometric parameters (Å, °)

**Table:**

O1—C9	1.2229 (16)	C1—C8	1.5355 (18)
O2—C8	1.2174 (16)	C1—C7	1.5364 (19)
N1—C4	1.3378 (18)	C2—C6	1.3913 (18)
N1—C5	1.3425 (18)	C2—C3	1.3942 (18)
N2—C9	1.3442 (17)	C3—C4	1.3897 (19)
N2—C1	1.4589 (16)	C3—H3	0.9500
N2—H2	0.8800	C4—H4	0.9500
N3—C8	1.3571 (17)	C5—C6	1.3884 (19)
N3—N4	1.4010 (16)	C5—H5	0.9500
N3—C9	1.4047 (17)	C6—H6	0.9500
N4—H4A	0.978 (19)	C7—H7A	0.9800
N4—H4B	0.96 (2)	C7—H7B	0.9800
C1—C2	1.5254 (18)	C7—H7C	0.9800
			
C4—N1—C5	116.49 (12)	N1—C4—C3	123.89 (13)
C9—N2—C1	112.63 (11)	N1—C4—H4	118.1
C9—N2—H2	123.7	C3—C4—H4	118.1
C1—N2—H2	123.7	N1—C5—C6	123.97 (13)
C8—N3—N4	122.57 (11)	N1—C5—H5	118.0
C8—N3—C9	112.46 (10)	C6—C5—H5	118.0
N4—N3—C9	124.96 (11)	C5—C6—C2	118.94 (12)
N3—N4—H4A	108.4 (11)	C5—C6—H6	120.5
N3—N4—H4B	112.4 (12)	C2—C6—H6	120.5
H4A—N4—H4B	106.2 (17)	C1—C7—H7A	109.5
N2—C1—C2	112.09 (10)	C1—C7—H7B	109.5
N2—C1—C8	101.43 (10)	H7A—C7—H7B	109.5
C2—C1—C8	112.65 (10)	C1—C7—H7C	109.5
N2—C1—C7	111.58 (11)	H7A—C7—H7C	109.5
C2—C1—C7	109.52 (11)	H7B—C7—H7C	109.5
C8—C1—C7	109.37 (11)	O2—C8—N3	126.83 (12)
C6—C2—C3	117.73 (12)	O2—C8—C1	126.77 (12)
C6—C2—C1	121.97 (11)	N3—C8—C1	106.38 (11)
C3—C2—C1	120.09 (11)	O1—C9—N2	128.95 (12)
C4—C3—C2	119.00 (12)	O1—C9—N3	123.97 (12)
C4—C3—H3	120.5	N2—C9—N3	107.08 (11)
C2—C3—H3	120.5		
			
C9—N2—C1—C2	121.59 (12)	N4—N3—C8—O2	−2.7 (2)
C9—N2—C1—C8	1.19 (14)	C9—N3—C8—O2	178.47 (13)
C9—N2—C1—C7	−115.16 (12)	N4—N3—C8—C1	178.74 (11)
N2—C1—C2—C6	−155.89 (12)	C9—N3—C8—C1	−0.11 (14)
C8—C1—C2—C6	−42.24 (17)	N2—C1—C8—O2	−179.19 (13)
C7—C1—C2—C6	79.71 (15)	C2—C1—C8—O2	60.81 (17)
N2—C1—C2—C3	29.50 (16)	C7—C1—C8—O2	−61.23 (17)
C8—C1—C2—C3	143.15 (12)	N2—C1—C8—N3	−0.61 (13)
C7—C1—C2—C3	−94.90 (14)	C2—C1—C8—N3	−120.62 (11)
C6—C2—C3—C4	−0.49 (19)	C7—C1—C8—N3	117.35 (12)
C1—C2—C3—C4	174.35 (12)	C1—N2—C9—O1	179.01 (13)
C5—N1—C4—C3	0.0 (2)	C1—N2—C9—N3	−1.30 (14)
C2—C3—C4—N1	0.4 (2)	C8—N3—C9—O1	−179.43 (12)
C4—N1—C5—C6	−0.4 (2)	N4—N3—C9—O1	1.8 (2)
N1—C5—C6—C2	0.4 (2)	C8—N3—C9—N2	0.86 (14)
C3—C2—C6—C5	0.1 (2)	N4—N3—C9—N2	−177.95 (11)
C1—C2—C6—C5	−174.61 (12)		

### Hydrogen-bond geometry (Å, °)

**Table:**

*D*—H···*A*	*D*—H	H···*A*	*D*···*A*	*D*—H···*A*
N2—H2···O2^i^	0.88	2.26	2.9184 (15)	131
N2—H2···N4^i^	0.88	2.69	3.526	159
N4—H4A···O1^ii^	0.977 (19)	2.139 (19)	3.0676 (16)	158.07
N4—H4B···O1^iii^	0.96 (2)	2.17 (2)	3.1003 (16)	162.23

Symmetry codes: (i) −*y*+5/4, *x*−1/4, *z*−1/4; (ii) *y*−1/4, −*x*+5/4, −*z*+1/4; (iii) −*y*+5/4, *x*+1/4, −*z*+1/4.
